# Curvotaxis directs cell migration through cell-scale curvature landscapes

**DOI:** 10.1038/s41467-018-06494-6

**Published:** 2018-09-28

**Authors:** Laurent Pieuchot, Julie Marteau, Alain Guignandon, Thomas Dos Santos, Isabelle Brigaud, Pierre-François Chauvy, Thomas Cloatre, Arnaud Ponche, Tatiana Petithory, Pablo Rougerie, Maxime Vassaux, Jean-Louis Milan, Nayana Tusamda Wakhloo, Arnaud Spangenberg, Maxence Bigerelle, Karine Anselme

**Affiliations:** 1 Université de Haute-Alsace, CNRS, IS2M, UMR 7361, Mulhouse, F-68100 France; 2 Université de Strasbourg, Strasbourg, F-67081 France; 3 Université de Valenciennes et du Hainaut Cambrésis, LAMIH, UMR-CNRS 8201, Le Mont Houy, Valenciennes, F-59313 France; 4 Univ Lyon, UJM-Saint-Etienne, INSERM, SAINBIOSE U1059, F-42023 Saint-Etienne, France; 5 Micropat SA, Côtes-de-Montbenon 30, CH-1003 Lausanne, Switzerland; 6 Laboratório de Biomineralização, Centro de Ciênça da Saúde, Federal University of Rio de Janeiro, Rio de Janeiro, 21941-902 Brazil; 7 Aix Marseille Univ, CNRS, ISM, Inst Movement Sci, Marseille, F-13288 France

## Abstract

Cells have evolved multiple mechanisms to apprehend and adapt finely to their environment. Here we report a new cellular ability, which we term “curvotaxis” that enables the cells to respond to cell-scale curvature variations, a ubiquitous trait of cellular biotopes. We develop ultra-smooth sinusoidal surfaces presenting modulations of curvature in all directions, and monitor cell behavior on these topographic landscapes. We show that adherent cells avoid convex regions during their migration and position themselves in concave valleys. Live imaging combined with functional analysis shows that curvotaxis relies on a dynamic interplay between the nucleus and the cytoskeleton—the nucleus acting as a mechanical sensor that leads the migrating cell toward concave curvatures. Further analyses show that substratum curvature affects focal adhesions organization and dynamics, nuclear shape, and gene expression. Altogether, this work identifies curvotaxis as a new cellular guiding mechanism and promotes cell-scale curvature as an essential physical cue.

## Abstract (web summary)

The effect that microscale surface curvature has on cell migration has not been evaluated. Here the authors fabricate sinusoidal 3D surfaces and show that the cell nucleus and cytoskeleton cooperate to guide cells to concave valleys in a process they coin curvotaxis.

## Introduction

In vivo, cells are evolving within complex three-dimensional (3D) environments that exhibit various topographical features, spanning several orders of size and organization. At the nanometric scale, cells are in contact with collagen fibrils and other protein polymers that compose the extracellular matrix (ECM). A large body of studies have highlighted that cells are sensitive to this scale of topographical organization. For example, seminal work from Dalby et al. have shown that cell can recognize nanometric pits on the substrate, and the organization of these pits can channel cell differentiation toward a specific lineage^1,2^. Nanometric grooves, nanotubes, or nanofibers of specific diameters that mimic the polymers found in the ECM have also been employed to control adhesion and differentiation of mesenchymal or neural stem cells^3–5^. In addition to these nanometric features, natural biotopes also exhibit larger topographical cues that are often curved and smooth, such as walls of blood vessels, bone cell cavities, acini, or other cell bodies.

The effect of cell-scale topographical architectures on cell behavior has been initially explored using a variety of microstructured surfaces such as microgrooves and micropillars^6,7^. It has been observed that cell-scale topographies could induce morphological changes^8,9^, migratory patterns^7,10,11^, as well as nuclear reorganization and cell differentiation^12,13^. For instance, microgrooves have been employed to polarize and mature cardiomyocytes, and reprogram fibroblasts into cardiomyocytes with a better efficiency than by using biochemical cues^14^. Although this research highlights the pleiotropic effect of cell-scale topographies, it is mostly based on geometrical model surfaces that are not representing the curved and smoothed cell-scale shapes encountered in vivo.

Pioneering work using glass tubes (constant convex curvature) shows that cells orient themselves along the line of minimal curvature, allowing them to minimize cytoskeletal deformation^15^. More recently, Song et al.^7^ have shown that T-cell migration is impacted by curvature, with cells migrating preferentially along concave microgrooves. In the same line, Werner et al.^16^ have used hemispherical structures to show that mesenchymal stem cells (MSCs) respond differentially to constant concave or convex curvatures, both in term of cell migration and differentiation. Finally, Bade et al.^17^ have shown that actin stress fibers in fibroblasts can be reorganized by curvature, affecting cell migration directionality.

Despite these recent efforts, our understanding of the specific impact of cell-scale curvature on cell behavior remains elusive and the involved mechanisms are unclear. Herein we develop a series of large edge-free cell-scale sinusoidal landscapes with minimized anisotropy and very low nanometric roughness, and employ these new model surfaces to investigate specifically the cell response to cell-scale curvature variations. We show that the cell-nucleus and cytoskeleton cooperate to guide the migrating cell toward concave valleys. In addition, substratum curvature affects focal adhesion (FA) dynamics, nuclear shape, and gene expression, demonstrating the important regulatory cue and its role in vivo investigated in more details.

## Results

### 3D sinusoids to probe the response to cell-scale curvature

**Fig. 1:**
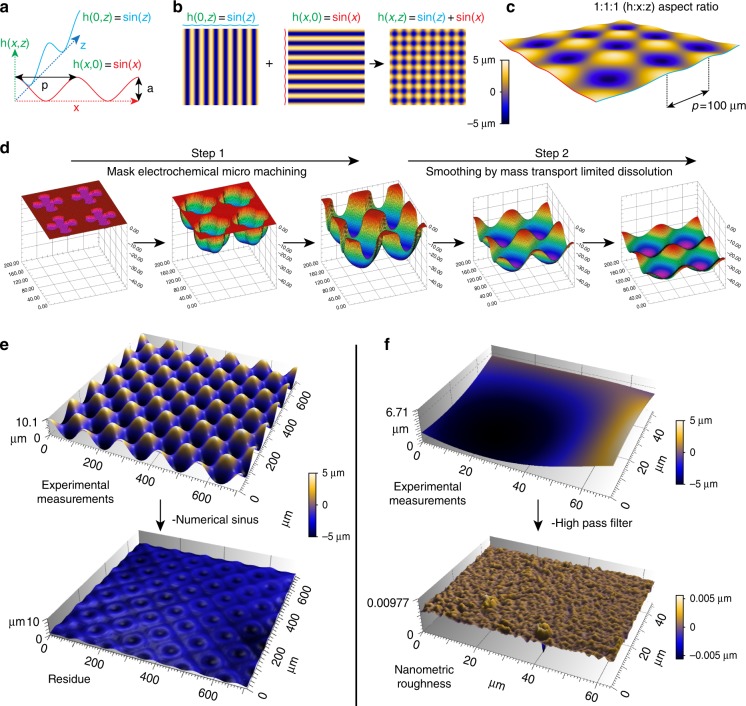
A two-step process for generation of ultra-smooth 3D sinusoidal topographies. **a**, **b** The surface can be described by the sum of two sinusoidal functions that are perpendicular to each other in a plane. **c** The image shows the result with 1:1:1 aspect ratio. **d** The fabrication process, simulated in **d**, consists in the creation of a preform through mask electrochemical micro machining (step 1) that is then smoothed out by mass transport-limited dissolution (step 2). Interferometric measurements (**e**, experimental) were compared to the corresponding numerical function. The residual topography (**e**, experimental minus numerical) shows that the surface is close to a sinusoidal function. Subtraction of long wavelength using a high-pass filter shows that the surface has a very low nanometric roughness (**f**)

A first major challenge was to create new model surfaces to probe specifically how cells react to cell-scale curved topography. This requires a fully curved surface deprived of topographic noise such as long-range anisotropy or nanometric roughness. We thus opted for a 3D sinusoidal model (Fig. 1 and Supplementary Figure 1). The shape of this surface can be described by the sum of two sinusoidal functions that are mutually perpendicular in the plane (Fig. 1a, b). The resulting function gives rise to an isotropic cell-scale hills-and-valleys array on which the cells are exposed to curvature gradients in all directions (Fig. 1c). To ensure the smoothness of the surface, we developed a two-step process in which angular 3D preforms were first created through mask electrochemical micromachining^18^ and then smoothened by mass transport-limited dissolution (Fig. 1d). Mask openings and dissolution charges were optimized by numerical simulations to reach a sinusoidal topography (Supplementary Movie 1 and 2, inspired by the work of West et al.^19^). Through this process, we generated a first series of 100 µm period surfaces with 10 µm amplitude (S10/100). To assess the geometrical quality of these surfaces, experimental measurements (interferometry) were compared to the corresponding closest mathematical functions (obtained by minimization). A graphical representation of a S10/100 surface before and after subtraction is shown in Fig. 1e. The flatness of the residual topography highlights the geometrical quality of the surface. Additional analysis reveals that the resulting stainless steel masters exhibit very low nano-roughness. Figure 1f shows an elemental surface before and after filtering for the determination of nano-roughness. The average *S*_a_ values and the corresponding standard deviation is below 1 nm (additional measurements are shown in Table 1). These metal templates were then replicated with polydimethylsiloxane (PDMS) to obtain a series of identical surfaces for experimentation.

**Table 1:** Topographical measurements for S10/100 and S5/100 surfaces

Name	Microrugosity (µm)	Period quality (µm)	Shape (µm)	Amplitude (µm)
S10/100	0.0004 ± 0.0001	141.7 ± 0.7	0.0842	4.96 ± 0.12
S5/100	0.0005 ± 0.0002	141.6 ± 0.9	0.0748	2.65 ± 0.06

### Mesenchymal cells position themselves on concave valleys

**Fig. 2:**
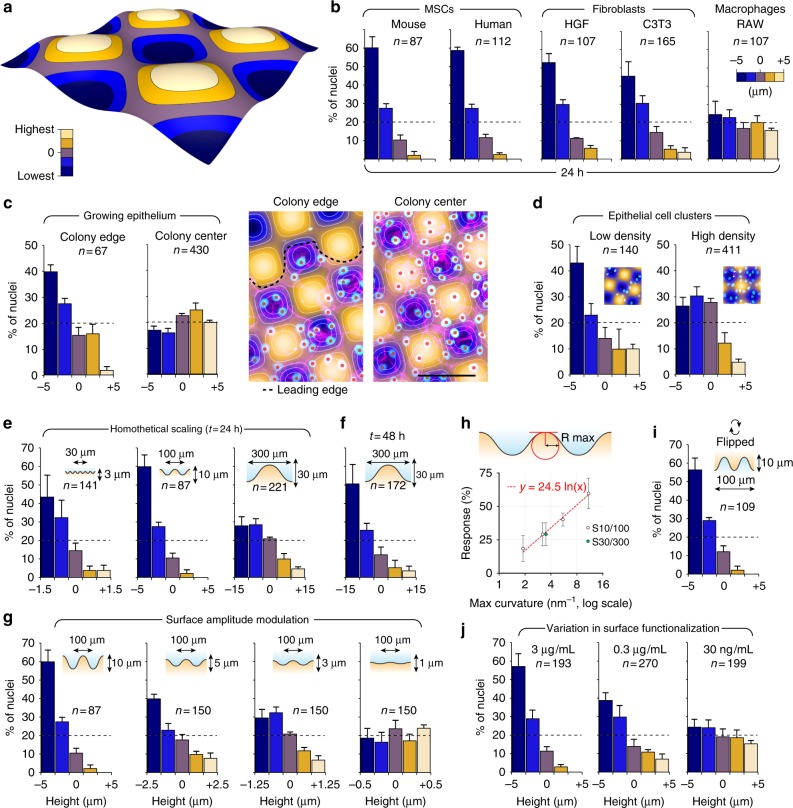
Adherent cells position themselves in the concave areas of cell-scale hills-and-valleys landscapes. **a** The surfaces were segmented into five areas delimited by isoheights contour lines. **b** Spatial distribution of different cell types assessed 24 h after surface inoculation on S10/100 surfaces. **c**, **d** Spatial distribution of MDCK cells within an epithelium (**c**) or forming small clusters (**d**). For the epithelium, the edge is compared with the center of the colony. **e**–**g** Surface distribution of mMSCs on homothetically scaled surfaces (**e**, **f**) and a surface series of decreasing amplitude (**g**). **h** A logarithmic relationship links the cell response, expressed in percentage of cells on concave, to the maximal curvature radius of the surface. **i** Cell response at 24 h when the surface has been flipped 2 h after cell adhesion. **j** Cell response on surfaces functionalized with decreasing concentrations of fibronectin. Error bars represent the standard error of the mean number of cells observed in three independent experiments. The total number of cells is indicated for each condition. Scale bar: 100 µm

We first screened a series of cell lines (MSCs, fibroblasts, macrophages, and epithelial cells) for their ability to respond to cell-scale curvature variations. As a screening criterion, we quantified their distribution with respect to surface topography 24 h after seeding, using nuclei as a position marker (Fig. 2). We segmented the surfaces into five height intervals (Fig. 2a) and counted the number of nuclei for each corresponding area. This first screen revealed that adherent cells (MSCs and fibroblasts) respond to surface topography by positioning their nucleus close to topographical minima, which correspond to the most concave areas of the surface (Fig. 2b). In regard to the topography, the strongest response was observed with MSCs (human and murine), which were not detected on convex areas, whereas macrophages were evenly distributed. Interestingly, epithelial cells within a growing colony exhibit a more complex pattern: in the leading edge, the cells tend to position their nuclei on concave topographies, whereas in the central part, their distribution is not impacted by curvature (Fig. 2c). This suggests that a progressive embedding into the epithelium modifies the cell response to topography. (Fig. 2d). Cells in small clusters also respond to topography, even for cell densities similar to those found within the epithelium. One simple explanation is that in small clusters or in the leading edge of a colony, cells have a high degree of freedom, whereas within a mature epithelium, cell–cell interactions might impose cell positioning regardless of the underneath topography.

### Biophysical parameters important for curvature sensitivity

We then tried to identify biophysical parameters important for cell sensitivity to curvature. Since MSCs exhibited the strongest response, we focused our analysis on this particular cell type. We developed a series of surfaces with various amplitudes and periods in order to define a threshold for curvature sensing. We designed homothetic surfaces with 30 or 300 µm periods, keeping the original 1:10 aspect ratio (S3/30 and S30/300). The shortest period tested—30 µm—still engages a strong response while the cells evolving on the longest one—300 µm—are less responsive over the same period of time (Fig. 2e). However, the distribution bias slightly increases after 48 h (Fig. 2f), suggesting a progressive cell positioning on concave. We also produced variants of the original S10/100 surface with 5, 3, or 1 µm peak-to-valley amplitude (S5/100, S3/100, and S1/100). We found that the cell response decreases with surface amplitude (Fig. 2g), following a logarithmic relation with the maximal curvature radius of the surface (Fig. 2h).

We also probed whether the bias in cell distribution was depending on gravity field direction. We let the cells adhere for 2 h, flipped the surfaces upside down, and quantified nuclear distribution 24 h after surface inoculation (Fig. 2i). We found that cells on inverted surfaces exhibit the same response as the ones with the regular surface orientation, demonstrating that cell response to topography does not depend on a passive sedimentation phenomenon.

Finally, we tested if the level of cell adhesion was an important factor. We functionalized the surfaces with decreasing concentrations of fibronectin while keeping the same incubation time, and assessed the protein density by infrared spectroscopy (Supplementary figure 2a). We found that the cell response to curvature decreases with ligand density, the response being negligible on surfaces presenting the lowest amount of fibronectin (Fig. 2j).

On surfaces with low ligand density, cells are compact with spherical nuclei, whereas those adhering on high ligand density are well spread with strongly compressed nuclei (Supplementary Figure 2b). Interestingly, cells on low ligand density present a similar morphology to macrophages (Supplementary Figure 2c and 2d), which are also unable to react to surface topography. This suggests that cell spreading and compression of the nucleus might play a role in the cell response to curvature.

Altogether, these results show that adherent cells are capable of sensing variations in cell-scale curvature and position their nuclei accordingly, the strength of the response increasing together with ligand density, time, and surface aspect ratio. Hereafter, we will refer to the mechanism allowing the cells to migrate toward negative curvatures as curvotaxis.

### Cell-nucleus vectors point toward negative curvature minima

**Fig. 3:**
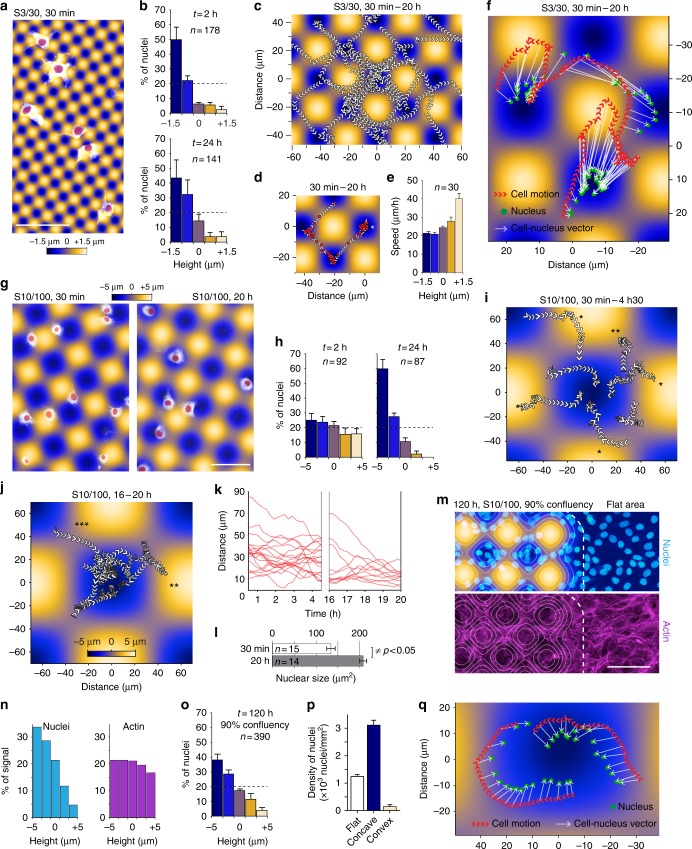
Curvotaxis allows adherent cells to avoid convex hills during their migration. **a**, **b**, **g**, **h** Cells labeled with cell tracker adhering on S3/30 (**a**) or S10/100 (**g**) surfaces and the distribution of their nuclei according to surface topography at the indicated time points (**b** and **h**). Nuclei are highlighted in red. **c** Tracking of nuclei on a S3/30 surface. Arrowheads indicate movement direction. **d** Tracking of a single nucleus over the same period. Positions are highlighted in red. **e** Average speed of nuclei according to surface height. **f**, **q** Tracking of cells and nuclei barycenters of cells moving on sinusoidal substrates. **i**, **j** Cell tracking at early (30 min–4 h 30 min) or late stages (16–20 h) of the interaction with the surface (*dodging event, **stopping event, ***migration through cols). **k** Distance between nuclei and surface minima over time, at early or late stages of the interaction. **l** Apparent nuclear size of cells adhering on S10/100 at the indicated time points. **m**–**p** Cells stained for DNA and actin on a S10/100 surface (**m**) and the height distribution of corresponding signal intensities (**n**), nuclei positions (**o**), and density of nuclei on flat, concave, or convex curvatures (**p**). Error bars in **b**, **h**, and **o** represent the standard error of the mean of three independent experiments. Error bars in **e** and **i** represent the standard deviation from the mean. The total number of cells is indicated for each condition. Scale bars: 100 µm

**Fig. 4:**
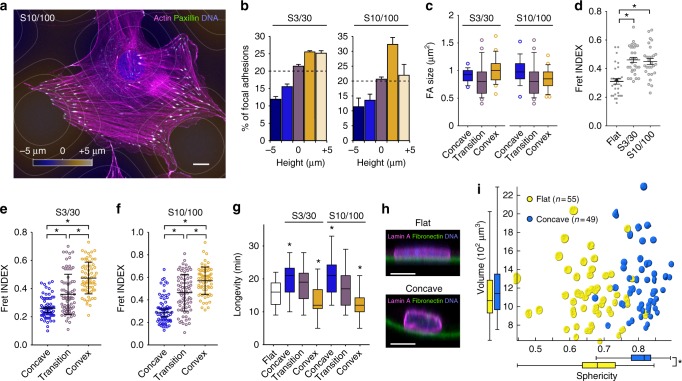
Cell-scale curvature affects focal adhesion organization and nuclear shape. **a**, **b** A cell stained for focal adhesions, DNA, and actin (**a**), and the distribution of focal adhesions according to surface height (**b**). **c**–**f** Impact of surface curvature on focal adhesion size (**c**), tension (**d**–**f**), and longevity (**g**). **h** Confocal imaging showing a cross section of the nucleus of mesenchymal stem cells immunostained for Lamin A on flat or concave surfaces labeled with fluorescent fibronectin. **i** Comparison of volume and sphericity of nuclei from cells growing on flat or concave surfaces (3D reconstructions from confocal data). The actual nuclear shape of each nucleus is displayed on the graph. *Significantly different from control using Student’s *t*-test with a *P* value < 0.05. Error bars in **b** represent the standard error of the mean. Error bars in **d**–**f** represent the standard deviation from the mean. Box plot elements in **c** and **g** display the minimum, first quartile, median, third quartile, maximum, and the suspected outliers. Scale bars: 10 µm

The accumulation of nuclei on concave areas could also originate from an impact of curvature on cell division rate. We performed a Ki67 staining (cellular proliferation marker) and did not find any correlation between marker intensity and surface curvature, suggesting a dynamic positioning mechanism (Supplementary Figure 3). To further test this hypothesis, we performed time-lapse imaging of cells evolving on S3/30 and S/10/100 surfaces on which we observed the strongest response. We first monitored cells migrating on the S3/30 surfaces. Cells are particularly responsive to this topography as they position their nuclei in the valleys from the beginning of the interaction (Fig. 3a, b). Cells explore dynamically their surroundings with peripheral protrusions while maintaining their nuclei close to the surface minima (see nuclei tracking in Fig. 3c, Supplementary Movie 3 and 4). We also observed that during their migration, cells quickly move their nucleus from valley to valley in a saltatory manner (Fig. 3d, Supplementary Movie 4). Nuclei velocities increase significantly during concave-to-concave transitions, their speed being significantly higher on convex topographies (Fig. 3e). Interestingly, cells lose their sensitivity to curvature just before dividing and regain it soon after they spread on the surface (Supplementary Movie 5). We also noticed that cell’s nuclei are always closer to the nearest valley than the center of the cell, the vector cell-nucleus always pointing toward surface minima (Fig. 4f, Supplementary Movie 4).

We then looked at cell migration dynamics on the S10/100 surfaces. At early time of the interaction, the cells are distributed randomly (Fig. 3g, h) and freely explore the surface (Fig. 3i, Supplementary Movie 6). We noticed that during their migration, cells manage to avoid convex bumps by modifying their trajectories or slow down when approaching the surfaces maxima (Fig. 3i). Later, cells tend to stabilize their position in concave regions (Fig. 3j, Supplementary Movie 7), their nucleus becoming closer to surface minima (Fig. 3k). At this stage, cells are well spread and interact strongly with the surface, as highlighted by the increase in apparent size of nuclei (Fig. 3l), and move only from valley to valley through topographical cols (Fig. 3j). Interestingly, when cells are entirely covering the surfaces, their nuclei remain excluded from convex areas (Fig. 3m–o), reaching three times higher densities than on flat areas (Fig. 3p), further suggesting a role for the nucleus in curvotaxis. Similarly, to what we observed on S3/30, the vector cell-nucleus is always orientated toward the closest surface minimum, suggesting that the nucleus is giving direction to the cell (Fig. 3q).

### Curvature affects FA organization and dynamics

We then looked at FA distribution on both S3/30 and S10/100 to seek for any correlation with surface topography. Although the distribution is variable from cell to cell, FA density seems maximal in the convex regions of the sinus (Fig. 4a, b), whereas FA size is not significantly impacted by curvature (Fig. 4c). We also investigated whether substratum curvature was affecting FA tension using a fluorescence resonance energy transfer (FRET) tension sensor (VinTS)^20^. In this construct, the vinculin head and tail domains are separated by a tensile chain peptide and flanked by FRET donor and acceptor fluorophores (mTFP1 and venus, respectively). Tension-dependent stretching of this molecule thus leads to a drop in FRET signal in a graded and dynamic way^21^. FA from cells on both on S3/30 and S10/100 surfaces are characterized by an overall lower state of tension (higher FRET index) compared to their flat control counterparts (Fig. 4d). However, the FA positioned on the concave part of those surfaces exhibit higher mechanical tension that the FA positioned on the convex part (Fig. 4e, f). We also looked at FA dynamics and found that FA longevity significantly increases on concave compare to convex curvatures (Fig. 4g). Together, these findings show that cells establish numerous but short-lived and less-tensed FA on convex curvatures, whereas their anchoring points to concave areas are subjected to higher intracellular tension and thus, get more stable. This suggests that curvature-dependent asymmetry in FA density and dynamics contribute to cell positioning on concave areas.

Since cells tend to position their nucleus on concave, we next asked how curvature might affect its shape. We grew MSCs on flat or concave surfaces, performed 3D reconstruction of their lamina and quantified nuclear shape and volume changes (Fig. 4h, i). We found that nuclei of cells positioned on concave surfaces become more spherical (sphericity index: 0.81) than those cultivated on flat (sphericity index: 0.67), whereas their average volume remained constant. This suggests that cell positioning on concave curvatures lead to a mechanical relaxation of the nucleus. When cells are in a concave region, the adhesions are more elevated. The stress fibers, which are connected to the substrate through the adhesions, might not compress the nucleus as much as on flat surfaces.

### Curvotaxis requires actomyosin dynamics, Lamin A, and Linkers of the Nucleoskeleton to the Cytoskeleton

**Fig. 5:**
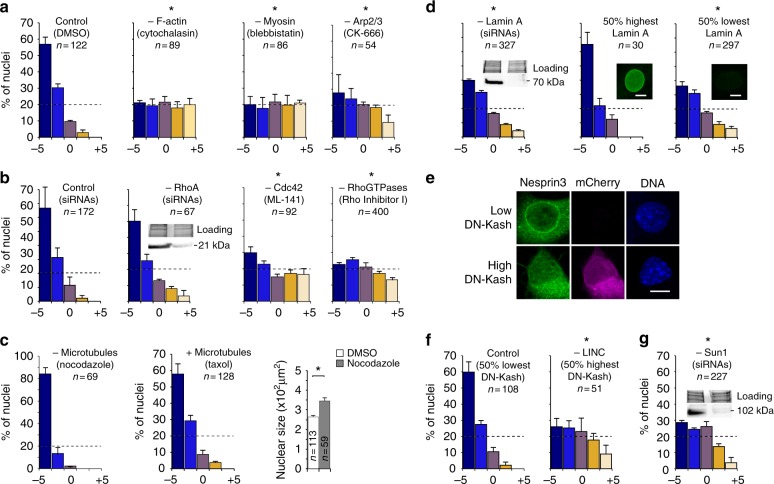
Curvotaxis relies on actin dynamics (**a**, **b**), high Lamin A expression (**d**), and functional LINC complexes (**e**, **f**, **g**) but not microtubules (**c**). Impact of various drugs, siRNAs, or dominant negative construct (DN-Kash) overexpression on height distribution of mouse mesenchymal stem cells 24 h after adhesion on 3D sinusoidal surfaces. Western blots with total protein input are shown as controls for siRNA efficiency (RhoA in **b**, Lamin A in **d**, and Sun1 in **g**). *Significantly different from control using Student’s *t*-test with *P* value < 0.05. Error bars represent the standard error of the mean number of cells observed within a given height interval in three independent experiments. Scale bars: 5 µm

To get more insights on the curvotaxis mechanism, we used a series of drugs or small interfering RNAs (siRNAs) targeting cellular components potentially involved in the cell response to topography (Fig. 5). We first assessed the importance of the actin network and its associated regulatory partners (Fig. 5, b). We found that drugs depolymerizing actin (cytochalasin) or blocking myosin II activity (blebbistatin) have a strong effect, leading to a homogeneous cell distribution. The actin-myosin network pulls on FAs, generating forces across the cell that tend to compress the nucleus on the surface^22^. Blebbistatin reduces cell spreading, stress fiber density, and lamellipodia formation^23^, leading to dendritic-like-shaped cells (Supplementary Figure 4a). Complete disruption of the actin network results in very low cortex tension and rounded nuclei (Supplementary Figure 4b). The actin polymerization regulator Cdc42 and the branched actin nucleator Arp2/3 complex are also essential for the response (Fig. 5b). Cdc42 is involved in the regulation of many processes related to cell migration, including filopodial protrusion formation^24^, cell polarity^25^, actomyosin contractility^26^, and FA assembly^27^. Arp2/3 is known to be necessary for lamellipodia formation and cell responses to ECM cues, including haptotaxis^28,29^. RNAi targeting RhoA do not have a strong impact on the cell response, possibly due to incomplete extinction at the protein level (Supplementary Figure 4c and 5b) or redundancy with other Rho isoforms. However, the use of a cell permeable inhibitor targeting all Rho GTPases (Rho Inhibitor I) showed that treated cells do not respond to topography anymore (Fig. 5b). This demonstrated that Rho activity is essential for curvotaxis. RhoA, B, and C play essential roles in cell migration, regulating the assembly of contractile actin-myosin filaments and FA^30^.

Interestingly, depolymerization of microtubules using nocodazole leads to an slight increase in the response, whereas microtubule stabilization has no effect (Fig. 5c). We also observed an increase in nuclear compression after depolymerization (Supplementary Figure 4b). Microtubules influence cell shape and mechanics by resisting to compressive forces exerted by the actin-myosin contractile network^31,32^. Their depolymerization could therefore result in an overall increase in compression, explaining the apparent flattening of nuclei. Alternatively, microtubule disruption could increase contraction by stimulating RhoA signaling and the mechanochemical activity of myosin^33–35^.

This first analysis shows that a fine tuning of actin dynamics is necessary for curvature-guided migration to occur, and strongly suggests that the actin-dependent compression of the nucleus is important for curvature sensing. The nucleus is approximately 5–10 times stiffer than the rest of the cell^36^ and this difference in stiffness might be important for curvotaxis to occur. To test this, we treated the cells with RNAi targeting Lamin A, the main contributor to nuclear stiffness^37^. Lamin A silencing significantly affected cells’ ability to respond to curvature (Fig. 5d). As an internal control, we looked at the fraction of treated cells that were still expressing high levels of Lamin A (Fig. 5d, Supplementary Figure 4f and 4g). We found that this subgroup was as responsive as untreated cells, confirming the correlation between Lamin A expression and curvature sensing. We also observed a direct correlation between cell positioning and Lamin A expression level (Supplementary Figure 4h and i). These data suggest that high lamin A levels are required for curvature-induced nuclear movements and thus cell migration during curvotaxis.

Intracellular displacement of the nucleus induced by its compression on curvature gradients explains well its positioning on S3/30 surfaces, on which concave minima can always be found underneath the adhering cell. However, on S10/100 and 30/300 surfaces, cells that adhere on convex curvatures have to migrate to position their nuclei on the closest concave minima, which is outside the cell body. When the nucleus moves within the cell, its interconnections with the cytoskeleton are likely to generate intracellular forces that might influence cell migration. We indeed observed stress fibers pointing toward the nucleus when it is strongly decentered (Supplementary Figure 4k), suggesting intracellular tensions induced by its positioning.

In living cells, Linkers of the Nucleoskeleton to the Cytoskeleton (LINC) are complexes bridging the nuclear lamina and the cytoskeleton and enabling force transmission within the cell through the nucleus^38,39^. They are formed by the interaction of SUN proteins that span the nuclear envelope and nesprin proteins that interact with cytoskeleton components. The N-terminal part of SUN proteins interacts with lamins and nuclear pore complexes whereas their C-terminal domain associates with the KASH domain of nesprins. To assess the importance of LINC complexes, we overexpressed a dominant negative (DN-KASH-mCherry) that saturates available binding sites on SUN proteins, resulting in the displacement of nesprins from the nuclear envelope into the endoplasmic reticulum (Fig. 5e). We found that overexpression of DN-KASH affects strongly the cell response to curvature, the cells with highest expression presenting the strongest phenotype (Fig. 5f). We also tested the role of LINC complexes using siRNAs against *SUN1* mRNAs (Supplementary Figure 4e). We found that the silenced cells display a similar phenotype as the ones overexpressing DN-KASH (Fig. 5g). These last results show that nucleo-cytoskeletal coupling plays a central role in curvotaxis.

### 3D sinusoids have distinct effects on hMSC transcriptome

**Fig. 6:**
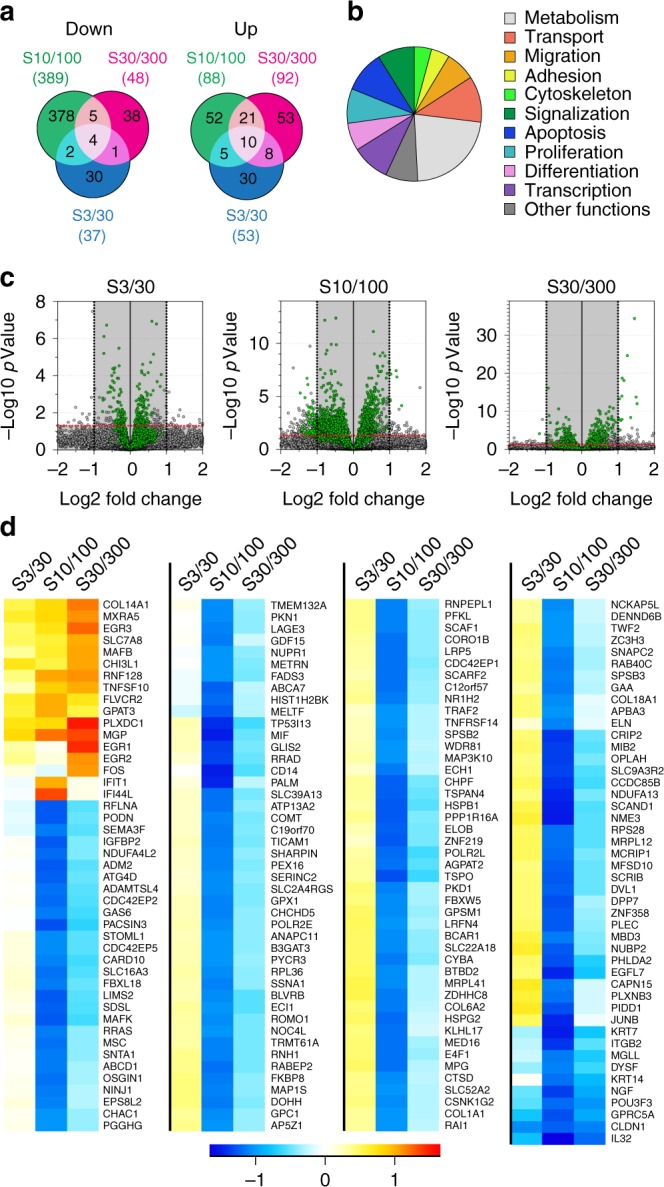
Sinusoidal homothetic surfaces have distinct effects on hMSC transcriptome. **a** Venn diagram representation of the total up- and downregulated genes (*P* value < 0.05 and Log2FC > 1) on sinusoidal surfaces using flat as reference (637 genes total). **b** Functional enrichment analysis using GO annotations of biologically described genes (361 genes). **c**, **d** Volcano plot of all differentially expressed genes filtering out low expressed genes (gray shaded area, reads < 100) (**c**), resulting in the identification of 181 genes for which relative expression patterns are shown for the different sinusoidal surfaces (**d**)

We then asked whether the sinusoidal surfaces, in addition to changes in nuclear shape, intracellular tensions, and FA homeostasis, could also affect gene expression. We compared the transcriptome of hMSCs cultivated for 5 days on the homothetic sinusoidal surfaces series (S10/100, S3/30, and s30/300), using flat surfaces as a reference. We identified 637 genes differentially regulated using standard cutoff (Fig. 6a). Among them, 361 genes were characterized with biologically described functions (functional enrichment shown in Fig. 6b), including 181 highly expressed genes (green dots in Fig. 6c). Most identified genes were found based on their downregulation on S10/100 surfaces (expression profiles shown in Fig. 6d). On the other hand, S3/30 and S30/300 surfaces have moderate effects on gene expression.

The most strongly downregulated genes found on S10/100 encode proteins expressed in differentiated tissues such as KRT7 (keratin found in simple epithelia) or PALM (paralemmin expressed in neurons) and transcription factors involved in differentiation processes (GLIS2: neuronal differentiation and kidney morphogenesis, CRIP2: differentiation of smooth muscle tissue, GPRC5A: embryonic development and cell differentiation, and NGF: neuron growth). Genes involved in response to stress, cytoskeleton remodeling, and cell proliferation are also among the most downregulated. Interestingly, a small subset of genes are overexpressed on S30/300. Among them, *EGR1*, *EGR2*, and *EGR3* are encoding transcription factors that regulate cellular programs of differentiation, growth, and response to extracellular signals. We also found several upregulated genes related to ECM remodeling (e.g.: *COL14A1*, *MXRA5*, *CHI3L1*, and *MGP*). These results suggest that sinusoidal surfaces have distinct impacts on the transcriptomic activity of hMSCs, S10/100 having the strongest effect by downregulating a large subset of genes associated with differentiation processes.

## Discussion

**Fig. 7:**
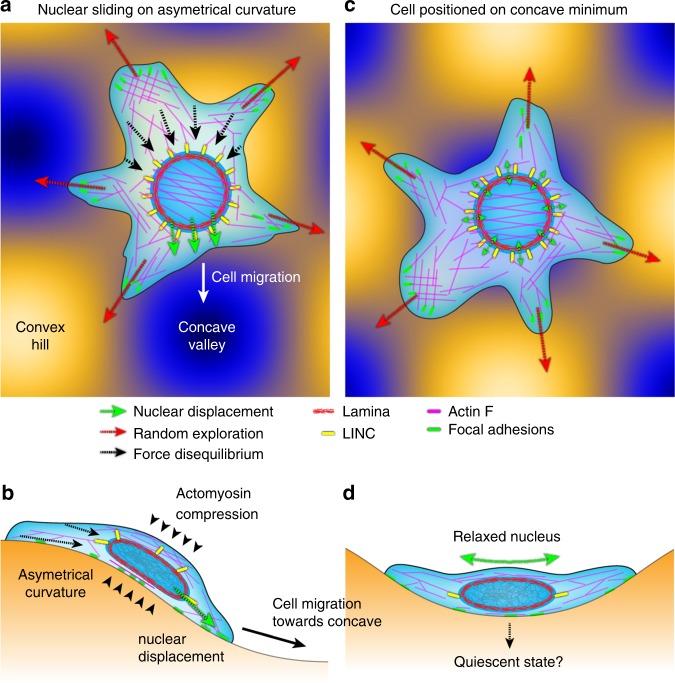
A model for curvature-guided cell migration. **a**, **c** Adhesion on asymmetrical curvature (**a**) triggers nuclear sliding toward concave, altering the random walk of the cell and leading to cell positioning on concave valley (**c**). **b**, **d** Side views showing actomyosin compression of the nucleus and its relaxation on concave curvature

Taken together, our data show that curvotaxis relies on the combination of two processes: surface exploration based on full actin dynamics and asymmetrical FA distribution and dynamics, and intracellular nuclear sliding triggered by actin-myosin-dependent cortical compression, the displacement of the nucleus giving direction toward the closest curvature minimum (Fig. 7a, b). Curvotaxis also requires LINC complexes, suggesting that nuclear sliding impacts the random walk of cells through mechanical coupling with the cytoskeleton. Contrary to other receptor-ligand-based directed migration mechanisms, curvotaxis is therefore a holistic phenomenon involving the whole-cell morphology and tensegrity. In fine, curvotaxis enables adherent cells to react to curvature variations and self-position in the most concave part of their substratum, leading to nuclear relaxation and changes in gene expression (Fig. 7c, d).

Cells can detect and respond to various environmental stimuli and modify their migrating trajectories accordingly. For instance, cells can integrate gradients of soluble factors and migrate toward higher concentrations through chemotaxis^40^. Many eukaryotic cell types can also detect gradients of molecules that are bound to the ECM (haptotaxis)^41,42^ or sense graded substratum rigidity (durotaxis)^43,44^ and migrate toward higher densities of ligand or stiffer areas. More recently, it was shown that cancer cells can also sense gradient of nanometric topographical cues via a process the authors called topotaxis, which relies on PI(3)K–Akt and ROCK–MLCK signaling pathways^45^. In these examples, the cellular sensing and integration mechanisms involved rely on ECM-triggered signaling. By contrast, we herein demonstrate the ability for adherent cells to respond to cell-scale curvature variations on their substratum through a mechanical module composed by the interplay of an actomyosin compressive cortex and a stiff nucleus. We thus extend our knowledge of the toolbox used by the cell to apprehend its environment. As a consequence, we may predict that cells progressing into curvy, fibrillary, or tubular environments will naturally follow the path of least nuclear mechanical stress. Therefore, it might be interesting to revisit previous observations of long-range cell migration and reinterpret them as a consequence of the topography itself rather than the existence of hypothetical chemical gradients^46^. Besides, ratchetaxis is a novel mode of directed migration in which the cell is not guided by a long-range gradient but by the repetition of cell-scale asymmetrical patterns (adhesive patches for example)^47^. In ratchetaxis and curvotaxis, the asymmetry impacts on the random walk of the cell by making a direction more favorable. Local asymmetry in the cell environment should therefore be considered as an important cue for directed migration and investigated in the in vivo context.

Considering the actomyosin/nucleus interplay as a mechanical sensor for curvature challenges how we usually conceive the role of these cellular components. The actin cortex and its interaction with the nucleus have already been implicated in cell polarization during migration-related processes^48^ and it is known that actin stress fibers are sensitive to local curvature^15,17^. However, the nucleus is often seen as a steric constraint limiting cell degrees of freedom and slowing down its movements, like in the context of confined migration^49^. In addition, recent data suggest that the nucleus is not necessary for establishing cell polarity or directional migration in two-dimensional but is important for cell response to mechanical cues and migration in 3D^50,51^. Assessing how enucleated cells react to curvature variations could be a way to clarify the effective contribution of other cellular components and reveal their role in nuclear-independent curvature sensing.

In our study, we show that cell-scale curvature modulations impacts cell migration, nuclear shape, intracellular tensions, and gene expression. The in vivo microenvironment present numerous curvature of cellular of supracellular scales. Glandular (acini) or tubular organs present typical curvature that may elicit a skewed migration of surrounding cells. During bone remodeling, the bone-degrading activity of osteoclasts creates cell-scale Howship lacunae, which are subsequently colonized by bone-producing bone-lining cells^46^. Similarly, extremity of long bones are made of hollow and bone marrow-filled trabecular bones. The behavior (migration or differentiation) of bone marrow progenitors or bone-remodeling cells may thus be instructed by the local curvatures of trabeculae. Matrix stiffness^52^, adhesive ligand densities^53^ or with specific nano-topographies^1,2^ produce similar effects to curvature. It would be interesting to determine whether these very different biophysical cues are integrated in fine through the same signaling hubs. The geometry of the nucleus imposes spatial and geometric constraints that encompass protein–DNA and protein–protein interactions^54^. It seems therefore particularly relevant to have a closer look at how changes in nuclear shape induced by cell-scale curvature affects the overall organization of chromosomal territories and modulates genome expression.

Altogether, this work establishes curvotaxis as a new cellular guiding mechanism. We propose that by affecting both cell migration and gene expression, cell-scale curvature should be considered an important regulatory cue and its role in vivo investigated in more details.

## Methods

### Engineering of 3D sinusoidal surfaces

3D sinusoidal surfaces were microstructured on 316L stainless steel coins (diameter 15 mm, thickness 1.5 mm) using a specifically designed two-step electrochemical process. The raw materials were first mechanically polished to obtain a mirror finish before being spin-coated with a polymeric resin (10 µm thick). The first process step mask pattern (diameter 8 mm) was then created through local laser ablation of the resin coating. Mass transport-limited electrochemical dissolution was performed under optimized hydrodynamic conditions. During this process the electrical charge flown through the set-up was constantly monitored. The experiment was stopped at a precise electrical charge corresponding to the desired dissolution depth. For the second process step, the remaining polymeric mask was laser ablated within a 10 mm diameter. The second electrochemical dissolution step was then applied until the final sinusoidal topography was reached. The geometric parameters of the first step mask and the electrical stop charges of both steps were optimized using numerical simulations (3D Laplace equation solver using a boundary elements method specifically implemented in Labview™ code). Two types of surfaces (S1 and S2) were micro-fabricated with a maximum amplitude of respectively 10 and 5 µm, both including 5027 peaks/valleys as expected for a sinus wave length of 100 µm on the two surface axes.

### Surface topography analysis and replication

Specimen topographies were measured using a 3D optical profilometer (Zygo NewView^TM^ 7300, Zygo Corp., USA) with a ×100 objective. The lateral resolution is equal to 0.22 µm and the vertical accuracy is about 1 nm. Measured surface areas are equal to 906 × 906 µm^2^ and are obtained by stitching 384 elemental surfaces of 69.5 µm × 52.1 µm. The measured surface is rectified using a polynomial of degree 1. The quality of the sine form is checked by minimizing the differences of shape between the experimental data and the mathematical function **Y** = *A* × (cos(**X**/*P* + phiX) × cos(**X**/*p* + phiZ)) where **X** is the abscissa of in-plane directions, **Y** is the computed height and the parameters determined by the minimization are the amplitude *A*, the parameter *P* proportional to the period, and phiX and phiZ are the phase shifts in the in-plane directions. The quality of the surface form is assessed by calculating the standard deviation of the height of the computed surface subtracted by the height of the measured surface i.e. the standard deviation of the residue. For microroughness quantification, elemental images are used in order to avoid stitching artifacts. Each elemental surface is rectified using a polynomial of degree 1 and then filtered using a high-pass Spline filter (ISO 16610–62) with a cutoff length of 5 µm. The arithmetic mean deviation *S*_a_ (ISO 25178) is computed for each surface and then the average and standard deviation of *S*_a_ are calculated. Curvature is visualized using a classical surface curvature measure: the mean curvature that is equal to the average of the principal curvature: **K**mean = ½ × (**κ**_1_ + **κ**_2_) where **κ**_1_ is the maximal curvature and **κ**_2_ is the minimal curvature.

Metal surfaces were first replicated by hot embossing of 35 mm polystyrene Petri dishes. Then, liquid PDMS (Sylgard^®^ 184) was casted inside the dishes to generate positive replicates (6 h incubation at 80 °C). The PDMS replicates were then coated with fibronectin (50 µM in phosphate-buffered saline (PBS)). Homogeneity of protein adsorption was verified by incubating fluorescent fibronectin on the surfaces and visualizing the coating by confocal microscopy.

### Imaging of cells and surfaces

Imaging was done on an upright Carl Zeiss LSM 700 confocal microscope (Germany), using ZEN software. For quantification of cell distribution, cells were fixed with 4% paraformaldehyde (Electron Microscopy Sciences, USA) for 10 min, labeled with Hoechst (5 µg/mL in PBS) for 10 min, extensively washed with 1× PBS and imaged using a ×20 dipping lenses in PBS. Surfaces were imaged in reflection mode with a 405 nm excitation laser. Topographical maps were generated from *Z*-stacks of the surfaces using TopoJ plugin (Compute Topography function) in ImageJ (NIH, USA). For live imaging, cells were stained with CellTracker™ Red CMTPX Dye (Thermoscientific, France) and *Z*-stacks were acquired at regular time intervals using a ×20 and an open pinhole (2 airy units) in an incubation chamber (Okolab, Italy) at 37 °C, 5% CO_2_, and H_2_O saturated. Surfaces were imaged at the end of the acquisition to reduce phototoxicity. Cell tracking was done on maximum projection *Z*-stack using Imaris software (Bitplane). For immunostaining, the cells were fixed with 4% paraformaldehyde for 10 min, permeabilized using 0.1% Triton X-100 for 5 min and blocked using 3% bovine serum albumin (BSA) for 30 min. Subsequently, the cells were incubated 1 h at room temperature (RT) with the appropriate primary antibody or fluorescent probe followed by incubation with respective secondary antibodies for 45 min and counterstained with Hoechst. 1× PBS washes were performed before each step. Primary antibodies or fluorescent probes used were Texas Red^®^-X Phalloidin (T7471, Molecular Probes, dilution 1:400), rabbit anti-Lamin A antibody (L1293, Sigma-France, dilution: 1:500), mouse Nesprin 3 antibody (ab123031, Abcam-UK, dilution: 1:200), and mouse anti-Vinculin hVIN-1 antibody (V9131, Sigma-France, dilution: 1:200). Secondary antibodies purchased from Abcam, UK used were goat anti-mouse (A-21151) or anti-rabbit (A-11008) conjugated with Alexa 488 (dilution: 1:400). For FA analysis, *Z*-stacks were processed using ImageJ software. Briefly, binary masks were created from max projections and quantified using particle analysis function with the corresponding topographical map as a reference. Image acquisition was done using a ×63 with optimal *Z*-sectioning parameters. For nuclear shape quantification, nuclear volumes were reconstructed from *Z*-stacks of Lamin A immunostained cells using Imaris suite software (all steps were performed at RT unless and otherwise stated).

### FA tension and dynamics

C3H10T1/2 cells were transiently transfected with the vinculin tension sensor VinTS^20^ (Addgene plasmid ID#: 26019). Transfected cells were plated at low density on sinusoidal surfaces and imaged using a Zeiss Axio-Observer Z1 inverted microscope. VinTS FRET channel images (excitation: 458 nm, emission: 533–587 nm), and VinTS donor channel images (excitation: 458 nm, emission: 469–501 nm) were obtained with a ×40 water-dipping objective (numerical aperture = 0.8). Signal from concave, convex, and transition areas were separated from *Z*-stacks and analyzed using FRET Analyser ImageJ plugin. FA were segmented using the water algorithm^55^. Briefly, each image was high-pass filtered using a round averaging of 10 pixels and segmented using the ImageJ morphological segmentation plugin MorphoLibJ. Contacts touching the edge of area of interest were systematically eliminated. Extremely low (<0.1) and high (>0.8) FRET indexes were not considered for the analysis as they can represent false-positive/-negative indexes. A minimum of 30 cells were recorded for each condition. For FA dynamics measurements, *Z*-stacks of VinTS donor channel were recorded every minute over a 45 min period. Maximal projections representing concave, convex, and transitional areas were send onto the Focal Adhesion Analysis Server^56^ to quantify the dynamics of FA. Dynamic parameter measurements were extracted after tracking of individual contacts using the software described in Berginski et al.^57^. The last version of the software is available on the Gomez lab website (http://gomezlab.bme.unc.edu/tools). Online analysis of FA dynamics can be done at http://faas.bme.unc.edu/.

### RNAi, overexpression, and drug treatments

Sequence-specific siRNAs were used to transiently knock down RhoA, Rac1, and Cdc42. We purchased MISSION^®^ Predesigned esiRNA for RhoA (EMU148751), Rac1 (EMU028841), CDC42 (EMU052411), and negative control #1 siRNA from Sigma-Aldrich. Briefly, cells at 60–70% confluency were transiently transfected with 50 or 100 nM siRNA (final concentration). After 6 h, the transfection mixture was removed and the cells were replenished with fresh Dulbecco’s modified Eagle medium containing 10% fetal bovine serum. The cells were then allowed to grow for the next 48 h prior to being used for subsequent experiments. The efficiency of RNAi treatments was assessed by quantitative PCR (qPCR). For DN-KASH overexpression, cells were cultured up to 60–70% confluency and then transfected with DN-KASH-mCherry plasmid (a kind gift from Dr. Nicolas Borghi, Jacques Monod Institute-France). Plasmid and RNAi transfections were carried out by using Lipofectamine 3000 (Thermoscientific, France) following the manufacturer’s protocol. For drug treatments, the optimum non-cytotoxic concentration was determined for each drug by standard MTT assay using 5 mg/mL thiazolyl blue tetrazolium bromide (Sigma, France). All the drugs were purchase from Sigma-Aldrich and employed using the following final concentrations: blebbistatine: 50 µM, cytochalasin D: 50 µM, rohdblock 6: 50 µM, nocodazole: 5 µg/mL, Paclitaxel: 50 nM, Rho inhibitor 1: 2 µg/mL, EHop0–16: 50 µM, CK-666: 100 µM, and ML141: 10 µM.

### Gene expression analysis

Total RNA was extracted from cell cultures reaching 50% confluency using the RNeasy Micro Kit (Qiagen) according to the manufacturer’s instructions. RNAs purity and concentration were evaluated using a Nanodrop spectrophotometer. For RNA-sequencing experiments, libraries were generated using TruSeq Stranded mRNA LT Sample Preparation Kit (Illumina). All samples were sequenced on the GenomEast genomic platform (IGBMC, Illkirch). Reads were mapped onto the hg38 of the *Homo sapiens* genome using STAR version 2.5.3a. Quantification of gene expression was performed using HTSeq version 0.6.1p1 with annotations coming from Ensembl version 91. Comparisons of interest were carried out using DESeq 1.16.1. Differentially expressed genes were first defined as those with an absolute log2FC ≥ 1 and an adjusted *P* value ≤ 0.05. Genes with a null raw read count were removed. The TSV file providing read counts for each genes together with *P* value and log fold-change was treated using FileMakerPro (version 14.0.1). For qPCR analysis, cDNAs were synthesized using the iScript^TM^ cDNA synthesis Kit (Bio-Rad) and 300 ng of total RNA. Real-Time PCR detection was carried out using SYBR Green reagents (Bio-Rad) on a CFX96 Touch^TM^ system (Bio-Rad) using standard procedures. Three reference genes (*gapdh*, *rplp0*, and *emgll*) were used to normalize the target gene expression and correct sample-to-sample variations. Primer sequences and corresponding gene accession numbers are listed in Table 2. Gene expression levels were analyzed based on the ΔΔCT method^58^.

**Table 2:** Nucleotide sequences of primers used for quantitative qPCR detection. “F” indicates the forward primer and “R” the reverse primer. *Gapdh*, *rplp0*, and *Emg1* were used for normalization

Gene name	Sequences (5′→3′)	Genbank accession number	qPCR product size (nucleotides)
*Gapdh*	F-TTCAACAGCAACTCCCACTC R-ATGTAGGCCATGAGGTCCAC	NM_008084.3	137
*Rplp0*	F-AACGGCAGCATTTATAACCC R-CGATCTGCAGACACACACTG	NM_007475.5	106
*Emg1*	F-ACGGCCCTCAGAAGCTATT R-TCACTACTGGGCACCAACTC	NM_013536.2	133
*RhoA*	F-CTCATAGTCTTCAGCAAGGACC R-GGCGGTCATAATCTTCCTGTC	NM_001313961	147
*Rac1*	F-TTATGACAGATTGCGTCCCC R-TGTCATAATCCTCTTGCCCTG	NM_009007	143
*Cdc42*	F-CATGTCTCCTGATATCCTACACAAC R-TGTCATAATCCTCTTGCCCTG	NM_009861	93
*Sun1*	F-CTGGGACGGTTCACCTATGA R-CCCGGAGCTCTACTATCTGGA	NM_024451	97
*LmnA*	F-CCGCTCTCATCAACTCCACT R-TCTCCATCCTCGTCGTCATC	NM_001111102	100

### Western blot analysis

Unless specified, instruments and supplies were all purchased from Bio-Rad (Germany). Western blots were performed according to standard procedures. Briefly, cell lysis and protein resuspension were achieved in radioimmunoprecipitation assay (RIPA) buffer. Then, total protein amount was estimated using the colorimetric Micro BCA™ Protein Assay (Thermoscientific, USA). Fifteen micrograms of total protein sample were loaded on a precast Mini-protean TGX stain-free gels and separated by electrophoresis. Protein gel images for normalization were acquired using ChemiDoc™ Imaging Systems before transfer. Proteins were transferred from the gel to 0.2μm polyvinylidene difluoride pre-activated membranes using the Transfer Blot turbo system. Afterwards, membranes were saturated with 3% BSA (Sigma, France) for 1 h at RT. Primary antibody incubation was performed overnight at 4 °C using the following antibody concentrations: Lamin A/C (1:2000, L1293, Sigma, France), Sun1 (1:1000, ab103021, Abcam-UK), and RhoA (1:500, 1B8-1C7, ThermoFisher Scientific). After short rinses, blots were incubated with the horseradish peroxidase secondary antibody (1:3000) for 1 h at RT. Protein bands were revealed using a chemiluminescent substrate (Clarity™ Western ECL Blotting Substrates) and digitally acquired using the ChemiDoc™ Imaging Systems. Relative protein levels were quantified using ImageJ software by normalizing the levels of knockdown proteins with total proteins. Uncropped blots are shown in Supplementary figure 5.

### Data analysis

Unless otherwise stated in the figure legend, the data presented herein are expressed as the mean ± standard error of the mean, the number of sample being displayed on the figures. Each experiment was repeated at least three times. Statistical analysis was performed using Student’s *t*-test and *P* values < 0.05 were considered significant, unless otherwise stated on the figure. All statistical tests and graphs were generated using Excel (Microsoft), DataGraph (Visual Data Tool), or Prism (GraphPad).

## Electronic supplementary material

Supplementary Information

Peer Review File

Description of Additional Supplementary Files

Supplementary Movie 1

Supplementary Movie 2

Supplementary Movie 3

Supplementary Movie 4

Supplementary Movie 5

Supplementary Movie 6

Supplementary Movie 7

## Data Availability

The data and materials generated and/or analyzed during the study are available from the corresponding author on reasonable request. The dataset related to the RNA-sequencing analysis that supports the findings presented Fig. 6 is available online using the following link: https://figshare.com/s/a18af5562974f4e63c44. The antibodies used in the study are the following: anti-Lamin A/C (1:2000, L1293, Sigma), Sun1 (1:1000, ab103021, Abcam), anti-RhoA (1:500, 1B8-1C7, ThermoFisher Scientific), and anti-Nesprin3 (1:1000, ab123031, Abcam). Validation statements are available on manufacturer’s websites (Sigma: https://www.sigmaaldrich.com, Abcam: http://www.abcam.com/, ThermoFisher: https://www.thermofisher.com/). C3h 10t1/2 and hMSC (StemPro™ BM Mesenchymal Stem Cells) cell lines were purchased from ThermoFisher.
