# Supplementary material for: Curvotaxis directs cell migration through cell-scale curvature landscapes
Source: Nat Commun. 2018 Sep 28;9:3995. doi: 10.1038/s41467-018-06494-6 (PMC6162274; doi:10.1038/s41467-018-06494-6)
Supplement: Supplementary file 1 — Supplementary Information [file 41467_2018_6494_MOESM1_ESM.pdf]

Curvotaxis directs cell migration through cell-scale curvature landscapes.

Pieuchot *et al*.

Supplementary Information

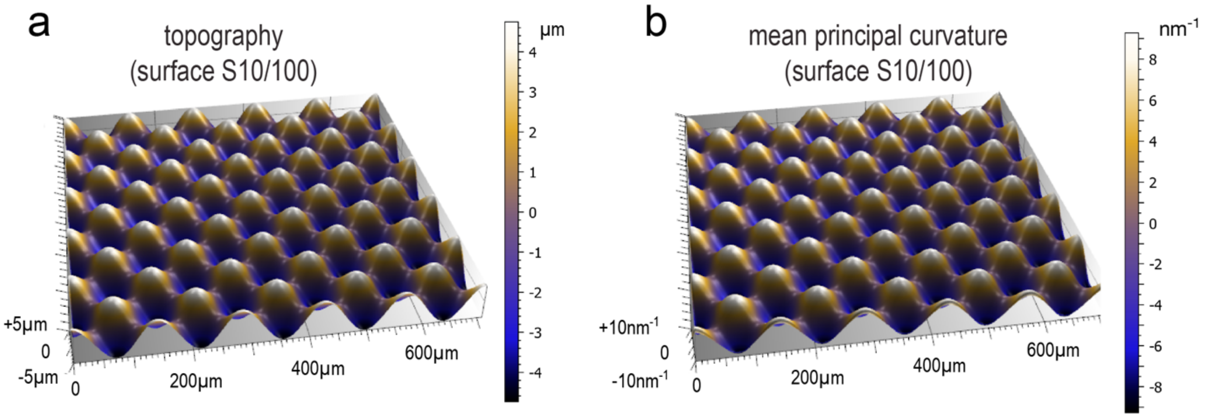

c

Surface	S3/30	S1/100	S3/100	S5/100	S10/100	S30/300
Period (µm)	30	100	100	100	100	300
Amplitude (µm)	3	1	3	5	10	30
Rho (µm)	22	912	313	182	92	283
Curvature max. (nm-1)	45,4	1,9	3,2	5,5	10,9	3,5

Supplementary figure 1. (a and **b).** Surface analysis showing a topographical map of the S10/100 surface (a) and the corresponding curvature map (b). Curvature is visualized using the mean curvature: $\mathbf{K}_{mean} = \frac{1}{2}*(\boldsymbol{\kappa}_1 + \boldsymbol{\kappa}_2)$ where $\boldsymbol{\kappa}_1$ is the maximal curvature and $\boldsymbol{\kappa}_2$ is the minimal curvature. (**c**) Numerical description of the sinusoidal surfaces used in this study.

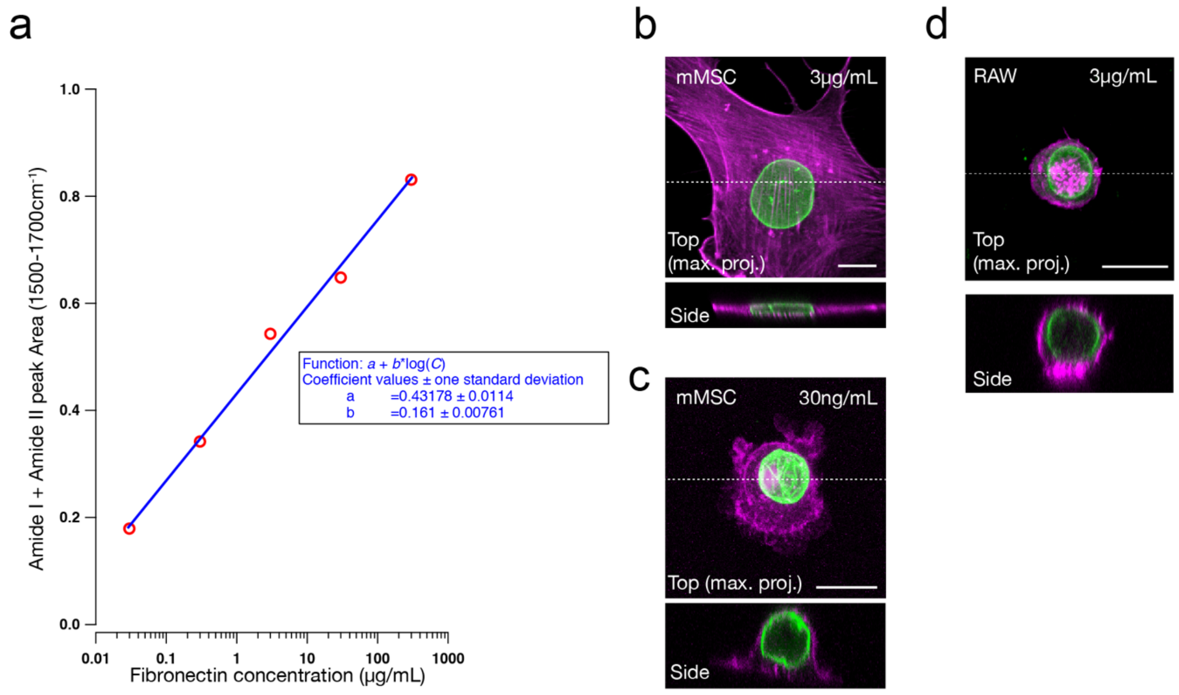

Supplementary figure 2. (a) Relation between amid peak intensity and fibronectin concentration. **(b** and **c)** Impact of ligand density on actin cytoskeleton (magenta) and nuclear lamina (green) on mMSC spreading. (**d**) Actin cytoskeleton (magenta) and lamina (green) of a macrophage (RAW cell). Scale bars: 10µm.

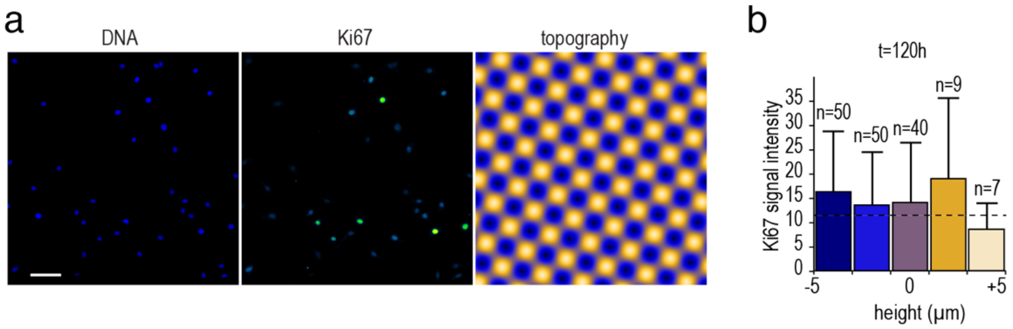

Supplementary figure 3. (a and **b)** Ki67 staining of cells on a S10/100 surface and quantification of the signal according to surface topography. Scale bar: 100µm.

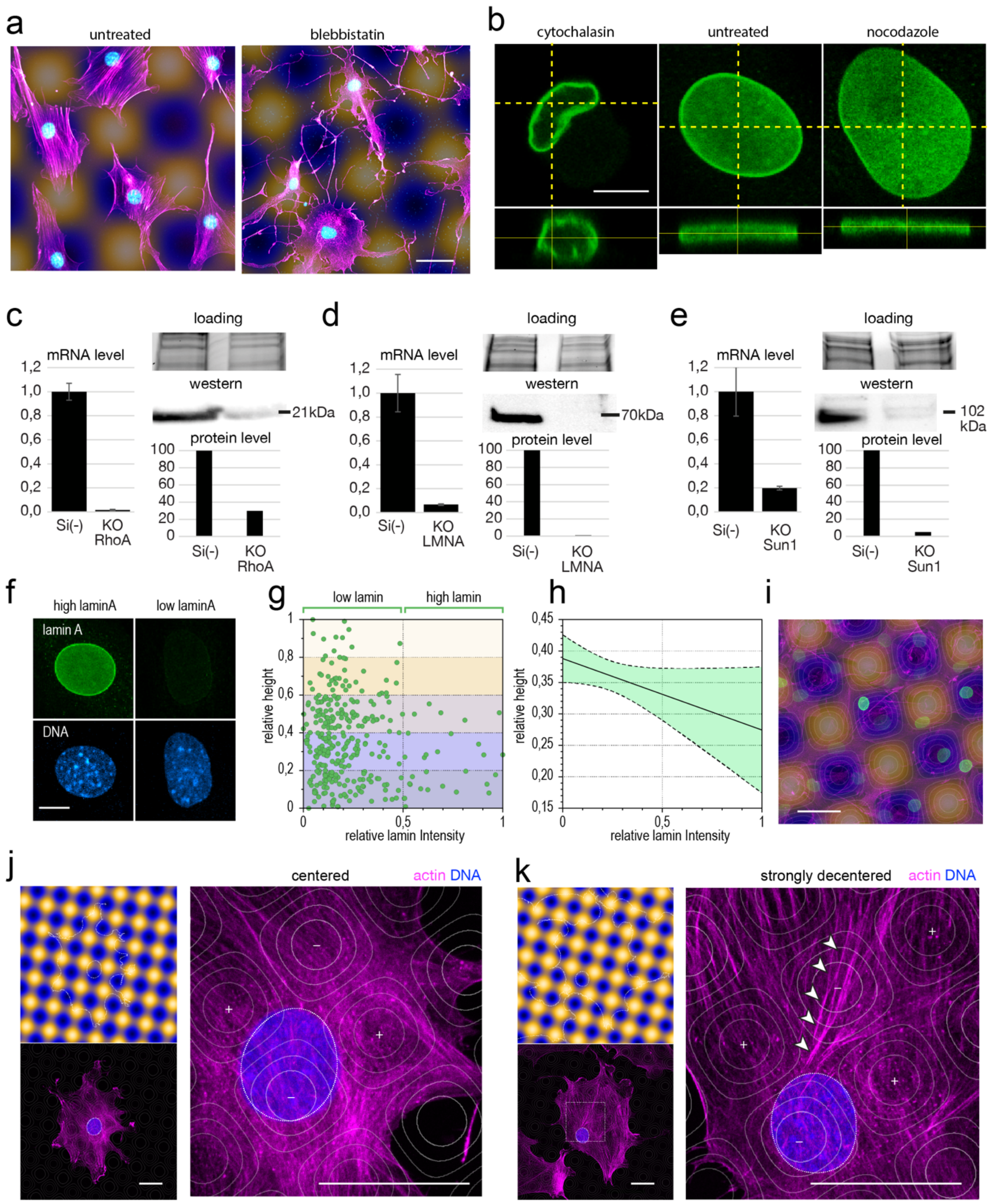

Supplementary figure 4. (a) Impact of blebbistatin on cells stained for actin. (**b**) Impact of cytochalasin and nocodazole on nuclear envelope morphology. (**c**, **d and e**) Impact of siRNA treatments on targeted mRNAs and proteins levels. (RhoA in **c,** LaminA in **d** and Sun1 in **e**). (**f**, **g**, **h and i**) Effect of siRNAs targeting laminA on cell distribution. Endogenous laminA

variations detected after immunostaining (**f**) is correlated with surface topography (**g**, **h** and **i**). (**j and k**) Immunostainings of cells adhering on S3/30 surfaces showing surface topography, DNA and the actin cytoskeleton. Arrowheads highlight actin stress fibers. Scale bars: 30µm in (a), 10µm in (b), 5µm in (f), 50µm in (i), 30µm in (j) and (k).

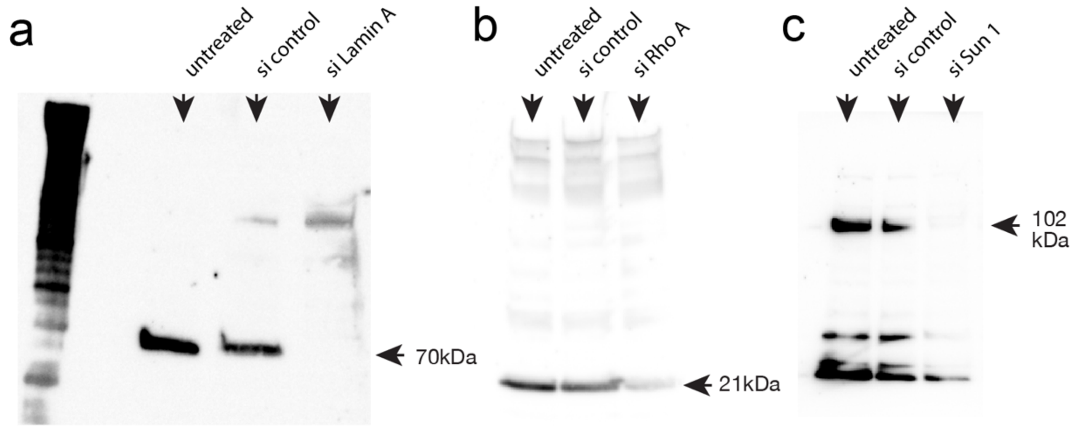

Supplementary figure 5. Full uncropped western blots showing Lamin A, Rho A and Sun 1 proteins from total protein extracts of untreated or RNAi treated C3H10 cells.
