# Supplementary material for: Curvotaxis directs cell migration through cell-scale curvature landscapes
Source: Nat Commun. 2018 Sep 28;9:3995. doi: 10.1038/s41467-018-06494-6 (PMC6162274; doi:10.1038/s41467-018-06494-6)
Supplement: Supplementary file 3 — Description of Additional Supplementary Files [file 41467_2018_6494_MOESM3_ESM.pdf]

Description of Additional Supplementary Files

File Name: Supplementary Movie 1
Description: Simulation of the first step of the sinusoidal surface fabrication process based on the creation of a 3D preform through mask electrochemical micro machining.

File Name: Supplementary Movie 2
Description: Simulation of the second step of the sinusoidal surface fabrication process that consists in the smoothing of the preform by mass transport limited dissolution.

File Name: Supplementary Movie 3
Description: Tracking of mouse mesenchymal stem cells migrating on a S3/30 sinusoidal surface. The topography is represented by a blue (concave) to yellow (convex) gradient.

File Name: Supplementary Movie 4
Description: Segmentation and tracking of mouse mesenchymal stem cells migrating on a S3/30 sinusoidal surface. The nucleus is coloured in red and the cytosol in white. The topography is represented by a blue (concave) to yellow (convex) gradient.

File Name: Supplementary Movie 5
Description: Tracking of a mouse mesenchymal stem cell migrating on a S3/30 sinusoidal surface. The cell divide during the time lapse (at time=500min). The topography is represented by a blue (concave) to yellow (convex) gradient.

File Name: Supplementary Movie 6
Description: Mouse mesenchymal stem cells migrating on a S10/100 sinusoidal surface 30 minutes after inoculation. The topography is represented by a blue (concave) to yellow (convex) gradient.

File Name: Supplementary Movie 7
Description: Mouse mesenchymal stem cells migrating on a S10/100 sinusoidal surface 16h after seeding. The topography is represented by a blue (concave) to yellow (convex) gradient.
