# Supplementary material for: Curvotaxis directs cell migration through cell-scale curvature landscapes
Source: Nat Commun. 2018 Sep 28;9:3995. doi: 10.1038/s41467-018-06494-6 (PMC6162274; doi:10.1038/s41467-018-06494-6)
Supplement: Supplementary file 2 — Peer Review File [file 41467_2018_6494_MOESM2_ESM.pdf]

Editorial Note: This manuscript has been previously reviewed at another journal that is not operating a transparent peer review scheme. The manuscript was considered suitable for publication without further review at Nature Communications.
